# “Is all the stuff about neurons necessary?” The development of lay summaries to disseminate findings from the Newcastle Cognitive Function after Stroke (COGFAST) study

**DOI:** 10.1186/s40900-017-0066-y

**Published:** 2017-09-11

**Authors:** Sarah Barnfield, Alison Clara Pitts, Raj Kalaria, Louise Allan, Ellen Tullo

**Affiliations:** 1 Newcastle upon Tyne Hospitals’ NHS Foundation Trust, Newcastle upon Tyne, UK; 2 Institute of Neuroscience, Newcastle University, Newcastle upon Tyne, UK; 3 School of Biomedical Sciences, Newcastle University, Newcastle upon Tyne, UK

**Keywords:** Stroke, Dementia, Dissemination, Lay summaries, COGFAST

## Abstract

**Plain English Summary:**

Why did we do this study?

It can be difficult for scientists to communicate their research findings to the public. This is partly due to the complexity of translating scientific language into words that the public understand. Further, it may be hard for the public to find out about and locate information about research studies. We aimed to adapt some scientific articles about the links between dementia and stroke into lay summaries to be displayed online for the general public.

How did we do it?

We collaborated with five people from a volunteer organisation, VOICENorth. They took part in two group discussions about studies reporting on the link between dementia and stroke, and selected four studies to translate into lay summaries and display on a website. We discussed the layout and language of the summaries and made adaptations to make them more understandable to the general public.

What did we find?

We were able to work with members of the public to translate research findings into lay summaries suitable for a general audience. We made changes to language and layout including the use of ‘question and answer’ style layouts, the addition of a reference list of scientific terms, and removing certain words.

What does this mean?

Working with members of the public is a realistic way to create resources that improve the accessibility of research findings to the wider public.

**Abstract:**

**Background**

Scientific research is often poorly understood by the general public and difficult for them to access. This presents a major barrier to disseminating and translating research findings. Stroke and dementia are both major public health issues, and research has shown lifestyle measures help to prevent them. This project aimed to select a series of studies from the Newcastle Cognitive Function after Stroke cohort (COGFAST) and create lay summaries comprehensible and accessible to the public.

**Methods**

We used a focus group format to collaborate with five members of the public to review COGFAST studies, prioritise those of most interest to the wider public, and modify the language and layout of the selected lay summaries. Focus groups were audio-taped and the team used the data to make iterative amendments, as suggested by members of the public, to the summaries and to a research website. We calculated the Flesch reading ease and Flesch-Kincaid grade level for each summary before and after the changes were made.

**Results**

In total, we worked with five members of the public in two focus groups to examine draft lay summaries, created by researchers, relating to eight COGFAST studies. Members of the public prioritised four COGFAST lay summaries according to the importance of the topic to the general public. We made a series of revisions to the summaries including the use of ‘question and answer’ style layouts, the addition of a glossary, and the exclusion of scientific jargon. Group discussion highlighted that lay summaries should be engaging, concise and comprehensible. We incorporated suggestions from members of the public into the design of a study website to display the summaries. The application of existing quantitative tools to estimate readability resulted in an apparently paradoxical increase in complexity of the lay summaries following the changes made.

**Conclusion**

This study supports previous literature demonstrating challenges in creating generic guidelines for researchers to create lay summaries. Existing quantitative metrics to assess readability may be inappropriate for assessing scientific lay summaries. We have shown it is feasible and successful to involve members of the public to create lay summaries to communicate the findings of complex scientific research.

**Trial registration**

Not applicable to the lay summary project.

**Electronic supplementary material:**

The online version of this article (doi:10.1186/s40900-017-0066-y) contains supplementary material, which is available to authorized users.

## Background

### Public understanding of science

Scientific research is often poorly understood by the general public and difficult for them to access. This presents a major barrier to disseminating and translating research findings. It is widely accepted that research should be disseminated to the public. Firstly, many research studies are funded by taxpayers in the UK via seven research councils and the National Institute for Health and Research (NIHR), part of the Department of Health [1]. As key stakeholders in research funding, the public have a right to expect findings to be communicated to them. Secondly, individuals often have an interest in specific research, especially if they are personally affected by the topic; understanding research findings may directly benefit them or their families through gaining relevant knowledge. In an era when the media can present confusing and conflicting messages about public health, it is important for the public to be able to access validated information that they can understand. Thirdly, the public may be more willing to contribute to ongoing research through a better understanding of what is involved and achieved. Accordingly, many grant applications now require evidence of plans to include members of the public in their research [2]. As of May 2014, the application form for NIHR funding must include a good quality plain English summary [3]. Improving the dissemination of research findings is one goal of a systematic process of “patient and public involvement and engagement” (PPI/E) in scientific research, increasingly advocated by research funders in the UK and elsewhere [4]. Co-creating lay summaries and other resources that are suitable for a general audience is one example of PPI activity that may facilitate the dissemination of research findings.

### How do we make research easily accessible and understandable?

Development of lay summaries are one solution to improve readability of research which might, in turn, facilitate public understanding [5, 6]. The “Natural Ground” project, established by the Association of Medical Research Charities (AMRC)to explore public and patient involvement (PPI) in allocating charity grants, found that lay people believed that academics were more likely to concentrate on areas of ‘exciting research’ in preference to those of public interest [7]. They also highlighted the need for better access to the results of research which should be written in plain English, including the use of lay summaries. Furthermore, they suggested that the public could be successfully involved in developing lay summaries, however this would require sufficient training, mentoring and continuous support for the lay people recruited.

Similarly, the “Patients Participate” project identified poor writing style and the use of scientific jargon as barriers to the public understanding of research. The project advocated for all UK authored research articles to have a lay summary, but noted that there was a need for the development of guidelines and templates for authors of research articles to use when developing lay summaries [8].

It is thus acknowledged that the development of plain English versions of research results, including lay summaries, is not necessarily easy. Firstly, preparation of such resources requires the allocation of additional time and funding. Secondly, translation of scientific language to plain English is complex, and there may be a need to produce a variety of formats to suit the diversity of the intended audience [7].

### Dissemination of lay summaries

Further to the production of lay summaries, members of the public also need to be able to access them. The internet has emerged as an important method for dissemination of health-related information [9]. The internet has advantages over other channels of dissemination; information can be accessed quickly, easily updated, and offer access to a wider audience than printed materials [9, 10]. However, this advantage is contingent on internet access which is not universal [11].

In this paper we report on the process of involving members of the public in the co-creation, improvement and dissemination of lay summaries in relation to the Newcastle Cognitive Function after Stroke (COGFAST) study.

## Methods

### Study setting

The Newcastle Cognitive Function after Stroke (COGFAST) cohort study is an example of biomedical research with findings of interest to the public and important public health messages. In the UK, one in six people over 75 will have a stroke at some point in their lifetime. Thereafter, they are at a nine-fold increased risk of developing post-stroke dementia [12, 13]. The Newcastle COGFAST cohort project aims to investigate why this risk is higher.

COGFAST recruited 415 older people with a stroke between 1999 and 2003. Patients were aged 75 or older at the time of their stroke and selected using hospital based stroke registers in the north east of England. Analysis of COGFAST data has confirmed that stroke survivors are at increased risk of developing dementia, and that there are modifiable risk factors such as stopping smoking and increasing physical activity levels which can reduce this risk [14]. Accordingly, it is important that COGFAST findings are understandable and accessible to the public.

### Aims

When COGFAST began in 1999, there was no time or funding allocated for the dissemination of the findings. The potential for COGFAST findings to prevent deterioration in brain function following stroke prompted the research team to try to effectively disseminate the findings to the public. The aims of this project were:

- To select and prioritise individual studies from the COGFAST project of relevance and interest to members of the public.
- To produce lay summaries relating to the selected studies.
- To make these lay summaries accessible to the public via a website.

We chose to define “lay people” in accordance with INVOLVE as, “neither academic researchers nor health or social care professionals” [15].

Step 1: Construction of first draft lay summary

The project team (SB, ET, LA, RK) short-listed eight COGFAST journal articles with a variety of important public health messages [14–22] with the intention to construct first draft lay summary for each. We reviewed external research websites to consider possible designs for the COGFAST lay summaries [23–25]. The most commonly used design features included small paragraphs and bullet points. The use of infographics was not commonplace. One website had a ‘quick read’ summary and another used a question and answer layout to convey information. We incorporated features from this range of websites to produce a variety of layouts for the eight COGFAST summaries as follows:

- Continuous paragraph.
- Question and answer layout with pictures.
- Question and answer layout without pictures.
- Question and answer layout with tables and graphs.

The project team collaborated to agree the content and terminology included in first draft lay summaries.

Step 2: First focus group

### Identification of volunteer members of the public

We chose to use focus groups as a format to discuss the project and review each COGFAST lay summary. An invitation to take part was distributed via a user group: VOICENorth (Valuing Our Intellectual Capital and Experience). VOICENorth is an organisation at Newcastle University with the aim of connecting with the public and involving them in university activity [26]. Their members range in age but mostly consist of retired members of the community residing in Newcastle. We invited members to take part in two focus groups, 1 week apart. The invitation included information about COGFAST and the aims of this particular project. Interested individuals replied via email with their contact details and a short description of why they were interested in taking part. Based on recommendations from the NIHR, Patients Participate and the Natural Ground project [1, 7, 8], we excluded individuals from an academic science background as they were not defined as lay people. We selected five volunteers to collaborate with, aiming to balance obtaining a range of views with the likelihood of facilitating an effective discussion. A brief description of the collaborators is outlined in Table 1.

**Table 1:** A description of collaborators attending the focus groups

Volunteer 1 is a male in his 70s whose mother died of Alzheimer’s disease. His daughter’s mother-in-law developed Lewy body dementia.
Volunteer 2 is a male in his 50s and had a stroke at the age of 39. His father has dementia.
Volunteer 3 is a male in his 70s who is a retired teacher. His wife is a manager of a care home, and his mother had a stroke leading to poor cognitive function. He also belongs to a writers group.
Volunteer 4 is a female in her 60s. Her father died with dementia and her mother had a stroke.
Volunteer 5 is a female in her 80s currently experiencing memory problems and took part to find out what she could do to improve her memory and how she can help others.

Volunteers 1, 2, 3 and 4 attended the first focus group. Volunteers 1, 4 and 5 attended the second focus group. Reasons that volunteers were unable to attend included illness, forgetting the session or other commitments.

### First focus group structure

We sent the first drafts of the eight COGFAST lay summaries via email to the collaborators 1 week before the first focus group. At the focus group, we also provided collaborators with a paper copy of each of the corresponding eight chosen COGFAST journal articles. An interviewer (SB) asked open-ended questions [27] to elicit thoughts on the lay summaries, in particular about the language and structure.

We distributed a feedback sheet to enable collaborators to provide written comments and to score each summary on its importance to them individually and the public in general (the feedback sheet is available as Additional file 1). The scale ranged from 1 to 10, with 1 signifying the least important and 10 being the most important. Questions included in the feedback sheet were based on the findings of the Natural Ground project [7]. Prompted by the scoring, the group discussed which four lay summaries they preferred to be taken forward for the study website, and why particular summaries should be excluded.

Step 3: Revision of first draft of 4 lay summaries

Based on the discussion and written feedback, the project team edited the first drafts of the four prioritised lay summaries and sent version two draftsback to the collaborators prior to a second focus group.

Step 4: Second focus group

Using a similar procedure to the first focus group, the four prioritised COGFAST lay summaries were presented to the group by the interviewer who sought collaborators’ suggestions for further improvement of language and structure. An additional activity in the second focus group involved the interviewer demonstrating a range of websites [23–25, 28–31] to illustrate examples of website formatting. Collaborators were asked about their preferences in terms of website layout.

Step 5: revision and assessment of readability

Based on the findings from the second focus group, the project team finalised the draft of the four lay summaries and developed a layout for the COGFAST website. In addition, in order to assess for readability of the summaries and evaluate change from first to final draft, the Flesch reading ease scale and the Flesch-Kincaid grade level was applied to the first and the final versions.

The 1949 Flesch reading ease scale [32] was adapted in 1975 by Kincaid and became known as the Flesch-Kincaid grade level [33]. The scores for these tools are derived from parameters such as average number of words per sentence and the average number of syllables per word. Text that is understandable to the public should aim to have a Flesch reading ease score of 60 or over (of a total possible score of 100) [34] - this is equivalent to Flesch-Kincaid reading grade of twelfth grade (US high school level, year 13/s year A-level UK equivalent). The Flesch Kincaid method is the earliest and most widely used method of assessing readability and has previously been applied to health information for patients [35]. Since then other readability tools have been developed such as Frys Readability graph, McLaughlin’s SMOG grading and the gunning FOG index, although there is a lack of consensus as to which is most appropriate for health related literature [36]. We chose the Flesch Kincaid method in this case due its simplicity, and availability through word processing software. We applied the Flesch scale and allocated a grade using Flesch-Kincaid to the first draft of each of the four prioritised COGFAST lay summaries. The scores were later compared to those relating to the final lay summaries.

Step 6: Dissemination via COGFAST website

Based on collaborators’ views about the websites examined during the second focus group, SB used a University owned software programme to create a COGFAST website.

### Ethical approval

In accordance with Health Research Authority guidelines we were working “with” the members of VOICENorth to co-create resources about prior research findings from the COGFAST project. As such, the volunteers were not research “participants” or “subjects”, rather they were part of a team contributing to the collaborative process of translation of academic journal articles to lay summaries [37]. As this constituted PPI activity rather than research, formal research ethics approval was not required. However, volunteers were aware that they were not obliged to take part and that they could retire from the project at any time. Volunteers provided written consent to audio-record the focus groups and for anonymous quotation to be published in our project report.

## Results

In order to demonstrate the evolution of the lay summaries, we include an example of one of the lay summaries at each stage of the process (Additional files 2, 3 and 4)

Step 1: Construction of first draft lay summary

The first drafts of the initial eight selected COGFAST studies were developed through collaboration between the project team and volunteer members of the public. An example first draft is available as Additional file 2.

Step 2: First focus group

Discussion in the first focus group concentrated on language and lay summary structure. Despite the project team’s attempts to use plain English for the first drafts, the collaborators commented there was too much jargon which made the lay summaries difficult to understand. They suggested that definitions should be less clinical:

“Is all the stuff about neurons necessary? Shrinkage is surely all that is required?”

“I was a bit worried about the word cohort because I think a lot of people who haven’t done statistical biology will not know what it is… if you replace it with ‘this is a collection of studies”

As a result some language was revised; however it was also advised by collaborators not to ‘dull down’ the text, for example:

“Replace getting dementia with developing dementia.”

In terms of presenting data, collaborators concluded that there was no need to quantify findings:

“I am not sure if numbers matter. If it is an increased risk, it is an increased risk… to ease lay people into it, quantifying it doesn’t add anything.”

“I think you need to have a statistical mind to understand what has been put down, you need to make it a little more user friendly.”

Consequently we changed the summaries to include less jargon and only use numbers when essential. Collaborators also suggested that most definitions should be removed from the lay summaries and put into a glossary of terms on the website.

The four example layouts used for the first drafts were presented to collaborators. By consensus they found the question and answer layout most appealing. In contrast, collaborators struggled with the continuous paragraph layout and suggested that text should be presented in small separate sections:

“A slab of text is difficult to concentrate on.”

“If it wasn’t in bite size pieces it didn’t stick in the same.”

Considering images, collaborators were more comfortable with pictures than graphs and tables. They agreed that graphs could be included if they were easy to interpret but that tables should be removed. As a result, we only retained a single graph within the eight summaries:

“Pictures can explain things better than words.”

“The Sun certainly wouldn’t put them (tables and figures) in and neither would the Times, I think that gives you a good indication that charts or whatever are not that helpful to the lay reader.”

The order of sections was discussed and collaborators indicated that aims should be placed earlier in the summary. Further, they endorsed the use of the Alzheimer’s Society template of including a “quick read” section [23].

“Put the aims up front so people immediately know what it’s about.”

“Include a short quick lay summary before the full lay summary.”

As a result, we created a quick summary and placed it at the start with the aims re-sited at the beginning of the full summary.

One of the aims of the first focus group was to prioritise four of the eight first draft lay summaries to be adapted and disseminated via the COGFAST website. Collaborators rated the eight studies on their importance, to them individually and as they perceived to the public in general. Some felt that they should be prioritised on usefulness, others on interest, depending on the reader’s circumstances. Despite the difference of opinion, the group agreed that it was still important to encapsulate both interest and usefulness in prioritisation and so the papers chosen were not necessarily those which were most important to the individual collaborators. Two of the summaries [14, 19] were selected as they were felt to be of interest to the public and two others [16, 17] because they identified modifiable lifestyle risk factors.

Step 3: Revision of first draft of 4 lay summaries

Based on the suggestions of the collaborators in the first focus group, the research team adapted the first drafts of the four prioritised lay summaries. The key changes in format and language made at this stage are summarised in Table 2. The second draft of the example lay summary is available as Additional file 3.

**Table 2:** Changes to the first drafts of COGFAST lay summaries

Replacement of jargon	
Omission of numbers	
Preparation of a separate glossary of terms	
Use of question and answer layout	
Inclusion of pictures	
Omission of graphs and tables	
Inclusion of a “quick read” section	

Step 4: Second focus group

Collaborators reviewed the four revised lay summaries. The group agreed that the lay summary headings were suitable as they were short and simple, but suggested a series of spelling and grammar adjustments. The collaborators re-emphasised the importance of colour, images and text in small sections to make the summary more engaging.

The second focus group also considered website design. Collaborators viewed a series of example websites to examine their layout [23–25, 28–31]. Based on the features of these example website and their own previous experience of using websites, they made the following suggestions for the COGFAST website:

- To construct the website such that additional COGFAST summaries could be added in the future.
- To include an introductory page to explain what the COGFAST cohort is.
- A glossary of terms labelled A-Z
- Lay summaries to be made available as a PDF to print.
- Scientific terms in the lay summaries to be hyperlinked to the glossary.
- ‘Research for scientists’ section to include website links to the original papers.
- To include a ‘contact us’ section to provide information for the public about how to donate their brain or participate in a study.

Step 5: revision and assessment of readability

On the basis of the discussion in the second focus group, the project team finalised the versions of the four lay summaries to be made available to the public on the COGFAST website. The final version of the example lay summary is available as Additional file 4.

Flesh reading ease scores and Flesch-Kincaid grade level of the lay summaries before and after the focus groups is shown in Table 3. A higher Flesch reading ease score corresponds to text which should be easier to understand. In contrast, the higher the Flesch-Kincaid grade level, the more complex the text is estimated to. Interestingly, the reading ease scores and grade level of three of the lay summaries were estimated to be more complex following the changes suggested by members of the public.

**Table 3:** Flesch reading ease and Flesch-Kincaid grade level of the first draft of each lay summary compared to the final version

Lay summary number	Flesch Reading Ease		Flesch-Kincaid Grade Level	
	First draft (before focus group)	Final version (After focus group 2)	First draft (before focus group)	Final version (after focus group 2)
1	80	75.5	5.2	6.5
2	69.9	62.6	6.2	8.9
3	69.7	78.5	9.7	6
4	67.2	57.3	8.9	8.5

The higher the Flesch reading ease score, the easier the text is to read. The higher the grade level, the higher the reading age

Step 6: dissemination via COGFAST website

The COGFAST website [38] incorporated the formatting and design preferences of the collaborators as listed above, and is now available for members of the public to access. There is scope to expand the website to link to external sites relating to stroke and to dementia, and to add to the collection of lay summaries.

## Discussion

### Prioritisation, adaptation and dissemination of lay summaries

We have shown it is feasible and successful to work with members of the public to prioritise and adapt lay summaries to communicate the findings of complex scientific research. The focus group format was an effective way to create useful lay summaries and an appropriate design for a website to effectively disseminate the information. The collaborators appeared to be relaxed and comfortable, which is known to facilitate a more successful exchange of ideas [39].

Conversations emerging from the focus groups directed a series of suggestions to improve the original drafts of the lay summaries – a number of these adaptations align with previous literature around the production of lay summaries. For example, our collaborators identified that whilst unnecessary complexity should be avoided, language should not be “dumbed down” too much. Similarly, it is known that attempts to form “plain English” run the risk of resulting in “poor English” [15]. Our collaborators also agreed that scientific language should be avoided where possible but, when essential, a definition could be included. An evident strength of involving members of the public in our project was their ability to identify jargon missed by the academic team due to their familiarity with scientific terms.

Collaborators were also able to make valuable suggestions as to appropriate formatting of lay summaries and of a website to display them. For example, they preferred headings containing questions rather than statements. Following on from concerns about complex scientific terms, the group stressed the importance of a glossary of terms as a separate section to the website. Surprisingly, we did not find a glossary of terms on similar websites. The collaborators suggested the glossary would allow readers to easily refer to definitions if they needed to. By contrast, if all terms were defined within the body of the lay summary, the group believed this would be cumbersome and repetitive – an important barrier to engagement with the content. Their insights are likely to have led to a more accessible collection of resources from the point of view of members of the public, rather than something designed solely by an academic team. To promote longevity, a further valuable conclusion from the focus group was that the website should possess enough flexibility to allow further lay summaries to be added and adapted.

### The applicability of existing measures of reading ease

We expected Flesch reading ease scores and Flesch-Kincaid grade levels would correspond with the subjective views of collaborators. However, members of the first focus group criticised the lay summary with the second highest reading ease score (71) for its complexity. This is likely to be due to the use of scientific terms which could not be identified by these scales. Comparison of scores between first drafts and final versions of the four prioritised lay summaries suggested that final versions were more complex than the originals. This paradoxical finding suggests that the Flesch reading ease and Flesch-Kincaid grade level alone are not reliable methods of judging the suitability of a scientific lay summary. Given that scientific research is complex and varies greatly in terminology, we conjecture that it would be difficult to create a specific template or quantitative assessment tool that would be suitable for this purpose. However, we endorse the use of existing general guidelines on the production of lay summaries in conjunction with input from members of the public.

### Limitations

We acknowledge a series of limitations. Our method of seeking volunteers meant that our collaborators were not necessarily representative of the general public. Firstly, the group were highly educated; collaborators themselves acknowledged that they may have a higher level of scientific knowledge than average. For example, one volunteer (a retired teacher who studied biology) conceded he could understand tables and graphs, whereas a lay person may not be able to. Throughout, collaborators attempted to take into account how other lay people might vary in their ability to interpret the text. Secondly, all the collaborators had prior experience of involvement in other scientific engagement activities through VOICENorth. Those with enthusiasm and interest in academic activity and dissemination, those with availability, and those with access to a computer are more likely to have volunteered. Finally, all the collaborators had personal experience of a relative or friend with dementia or stroke. As a result, their suggestions may have influenced the type of language used in lay summaries. For example, one volunteer expressed that the language needed to more heartfelt.

Consequently, our group of collaborators may not be representative of a typical ‘lay person’ (if such a thing exists). However, we argue that these individuals are representative of those most likely to access and benefit from the COGFAST website. It may be that purposive sampling of members of the public who comprise the most likely audience of a dissemination strategy is more fruitful than random sampling.

Due to the time and funding constraints of the project, there were only two focus groups lasting 90 min each. This limited the time for discussion – not all the collaborators were able to contribute all of their suggestions. Further, some contributed more than others during the focus groups. To avoid the undue influence of more vocal collaborators, the feedback form allowed team members to offer written reflections to complement the verbal discussion. An alternative method to improve the data produced might be to use one-to-one interviews to allow uninhibited, in-depth conversation, although this might limit the achievement of consensus of views.

One way to facilitate future improvement to the COGFAST summaries would be to include an option for lay readers to provide feedback on the website, as recommended by Patients Participate [8], to allow for further editing. Lastly, we acknowledge the inherent flaw of using the internet to disseminate lay summaries given a proportion of the public (approximately 11% of households) are not online [11]. An alternative may be a COGFAST newsletter sent in the post with the lay summaries included or publicising the summaries elsewhere, for example, within VOICENorth’s regular newsletter.

Whilst this project used focus groups to develop lay summaries and design a website, there are supplementary methods that could have been used to improve the usability of the lay summaries and COGFAST website. The concept of “usability testing” is becoming more prevalent in response to the increasing use of the internet as a way of communicating. Essentially, any information presented via a website must meet the needs of its intended audience which requires planning, testing and amending (user research). The US government has established a website providing thorough guidance on website usability and the basics of user research [40]. Focus groups are cited as one potential method for understanding user needs, but there are other approaches such as “parallel design” and application of “System Usability Scales” (SUS) that could be adopted to make the COGFAST website more accessible than its current format [41].

## Conclusion

Involving members of the public in the collaborative prioritisation and revision of a series of lay summaries in relation to the COGFAST study led to iterative improvements of the initial drafts prepared by the academic team (see Additional files 2, 3 and 4). We suggest that the co-creation of scientific lay summaries should not rely solely on existing general guidelines, nor that they be assessed using existing measures of reading ease. Rather, that collaboration with members of the public to revise and refine content and layout of lay summaries is more likely to achieve the desired outcome of readability and comprehension.

## Additional files

Additional file 1: Feedback sheets used by collaborators in focus group 1 – Tuesday 9th February 2016. (PNG 93 kb)

Additional file 2: First draft of lay summary 3 – Constructed prior to and presented to focus group 1- Tuesday 9th February 2016. (ZIP 191 kb)

Additional file 3: Second draft of lay summary 3 – Completed after final focus group 1- Tuesday 16th February 2016. (ZIP 166 kb)

Additional file 4: Final version of lay summary 3 – Completed after focus group 2 – 26th February 2016. (PDF 429 kb)

## References

[1] National Institute for Health Research (2016). About NIHR.

[2] Auckland S (2010). Involving users in the research process. National Institute for Health Research.

[3] Involve. National Institute for Health Research. Plain English summaries in National Institute for Health Research (NIHR) funded research. 2017. http://www.invo.org.uk/plainenglishsummaries-newsletter/. Accessed 18 Mar 2017.

[4] Involve. National Institute for Health Research. About Involve. 2017. http://www.invo.org.uk/about-involve/. Accessed 18 Mar 2017.

[5] Kuehne LM, Olden JD (2015). Opinion: lay summaries needed to enhance science communication. Proc Natl Acad Sci U S A.

[6] Denegri S, Faure H (2013). It’s plain and simple: transparency is good for science and in the public interest. Trials.

[7] Association of Medical Research Charities (2009). Natural ground paths to patient and public involvement for medical research charities.

[8] JISC (2012). Patients participate! Workshop report.

[9] Shiovitz S (2011). Dissemination of quality-of-care research findings to breast oncology surgeons. Journal of Oncology Practice.

[10] Duffy M (2000). The internet as a research and dissemination resource. Health Promot Int.

[11] Office for national statistics: Internet access – households and individuals: 2016. http://www.ons.gov.uk/peoplepopulationandcommunity/householdcharacteristics/homeinternetandsocialmediausage/bulletins/internetaccesshouseholdsandindividuals/2016. Accessed 18 Aug 2016.

[12] Savva GM, Stephan BC (2010). Epidemiological studies of the effect of stroke on incident dementia: a systematic review. Stroke.

[13] Kokmen E (1996). Dementia after ischemic stroke: a population-based study in Rochester, Minnesota (1960–1984). Neurology.

[14] Allan LM (2011). Long term incidence of dementia, predictors of mortality and pathological diagnosis in older stroke survivors. Brain.

[15] Involve. National Institute for health and Research. Jargon Buster. 2015. http://www.invo.org.uk/resource-centre/jargon-buster/?letter=L. Accessed 4 Mar 2016.

[16] Firbank MJ (2012). Neuroimaging predictors of death and dementia in a cohort of older stroke survivors. J Neurol Neurosurg Psychiatry.

[17] Gemmell E (2012). Hippocampal neuronal atrophy and cognitive function in delayed poststroke and aging-related dementias. Stroke.

[18] Stephens S (2004). Neuropsychological characteristics of mild vascular cognitive impairment and dementia after stroke. Int J Geriatr Psychiatry.

[19] Allan LM (2013). Long-term incidence of depression and predictors of depressive symptoms in older stroke survivors. Br J Psychiatry.

[20] Morris CM (2011). NOS3 gene rs1799983 polymorphism and incident dementia in elderly stroke survivors. Neurobiol Aging.

[21] Ballard CG (2004). APOE epsilon4 and cognitive decline in older stroke patients with early cognitive impairment. Neurology.

[22] Gemmell E (2014). Neuron volumes in hippocampal subfields in delayed poststroke and aging-related dementias. J Neuropathol Exp Neurol.

[23] Alzheimer's Society. 2016. https://www.alzheimers.org.uk/. Accessed 4 Mar 2016.

[24] Stroke. Research. 2016. https://www.stroke.org.uk/research. Accessed 5 Mar 2016.

[25] Cognitive Function & Ageing Study (2016). What are the cognitive function and ageing studies.

[26] Newcastle University. Voice North. 2016. http://www.voicenorth.org/about-us/. Accessed 19 Nov 2016.

[27] Krueger RA, Casey MA (2015). Focus groups: a practical guide for applied research.

[28] University College London (2016). Patients.

[29] National Stroke Association (2016). Medical risk factors.

[30] Canadian Study of Health and Aging. 2002. http://www.csha.ca/. Accessed 4 Mar 2016.

[31] Canadian Study of Health and Ageing (2002). Introduction to the CSHA.

[32] Flesch R (1949). The art of readable writing.

[33] Kincaid JP (1975). Naval technical training command.

[34] Office for National Statistics (2015). Reading age.

[35] Cooley ME (1995). Patient literacy and the readability of written cancer educational materials. Oncol Nurs Forum.

[36] Badarudeen M, Sabharwal S (2010). Assessing readability of patient education materials: current role in Orthopaedics. Clin Orthop Relat Res.

[37] Health Research Authority/INVOLVE. Public involvement in research and research ethics committee review. 2016. http://www.hra.nhs.uk/documents/2016/05/hra-involve-updated-statement-2016.pdf. Accessed Apr 2017.

[38] Newcastle University. 2016 https://research.ncl.ac.uk/cogfast/home/. Accessed Mar 2017.

[39] Morgan DL (1997). Planning focus groups.

[40] US Department of Health and Human Services. Usability.gov. https://www.usability.gov/. Accessed Mar 2017.

[41] US Department of Health and Human Services. Usability.gov. https://www.usability.gov/what-and-why/user-research.html. Accessed Apr 2017.

