# Supplementary figures and images for: “Is all the stuff about neurons necessary?” The development of lay summaries to disseminate findings from the Newcastle Cognitive Function after Stroke (COGFAST) study
Source: Res Involv Engagem. 2017 Sep 11;3:18. doi: 10.1186/s40900-017-0066-y (PMC5611656; doi:10.1186/s40900-017-0066-y)

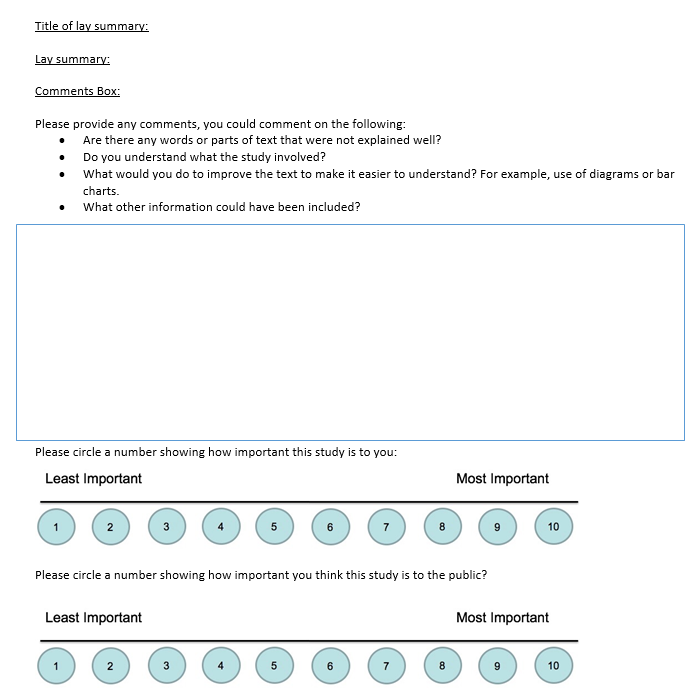

Supplement: Supplementary file 1 — Feedback sheets used by collaborators in focus group 1 – Tuesday 9th February 2016. (PNG 93 kb) [file 40900_2017_66_MOESM1_ESM.png]

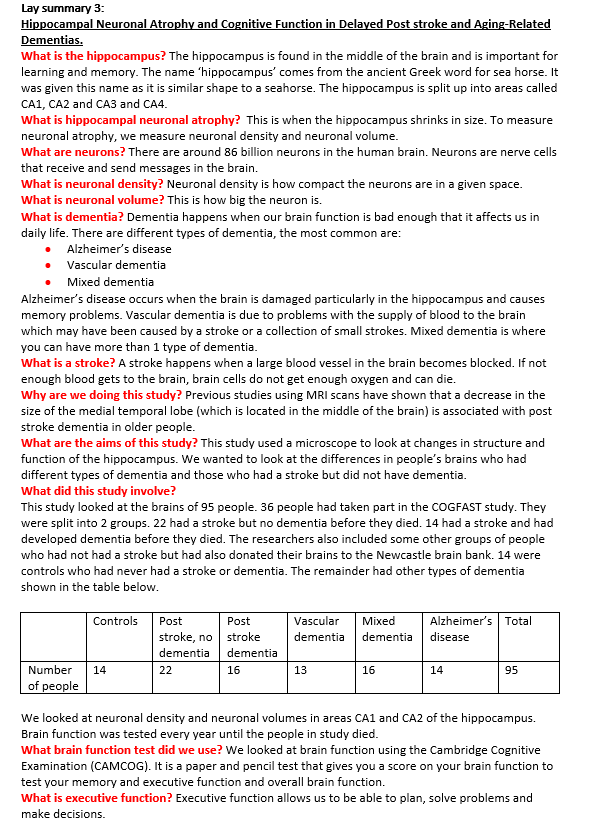

Supplement: Supplementary file 2 — First draft of lay summary 3 – Constructed prior to and presented to focus group 1- Tuesday 9th February 2016. (ZIP 191 kb) [file 40900_2017_66_MOESM2_ESM.zip › additional file 2/First draft of lay summary - side AR2.png]

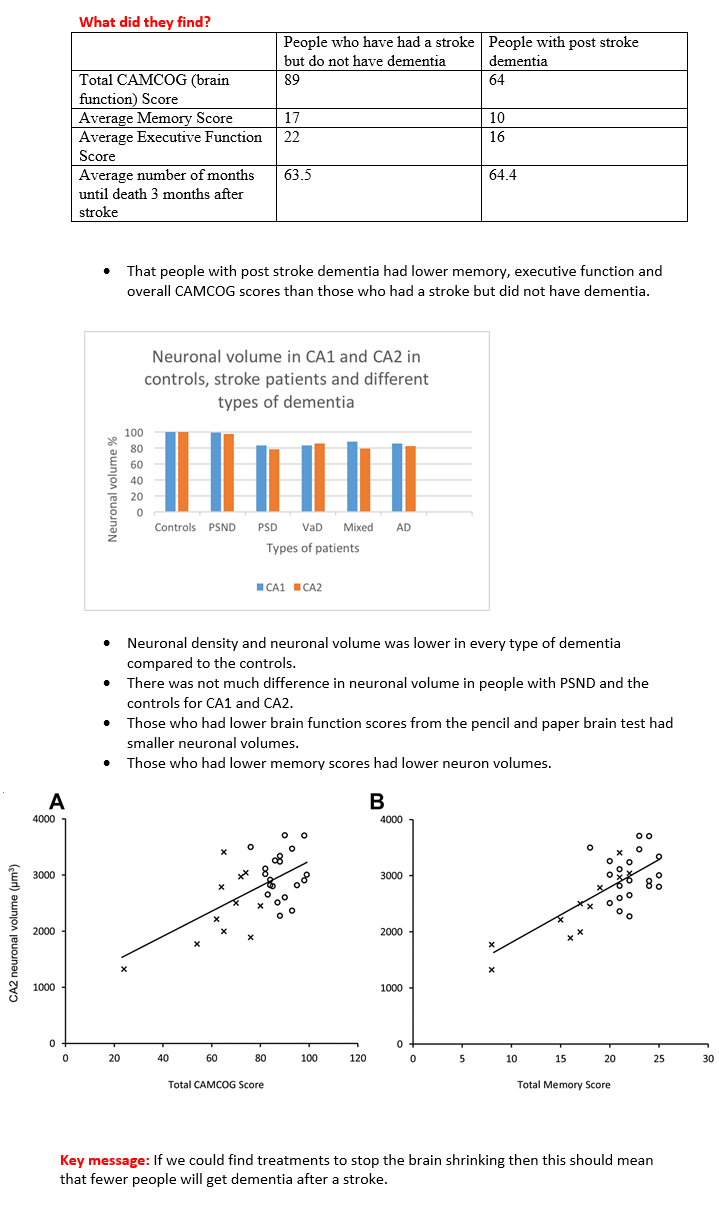

Supplement: Supplementary file 2 — First draft of lay summary 3 – Constructed prior to and presented to focus group 1- Tuesday 9th February 2016. (ZIP 191 kb) [file 40900_2017_66_MOESM2_ESM.zip › additional file 2/First draft of lay summary - side BR2.png]

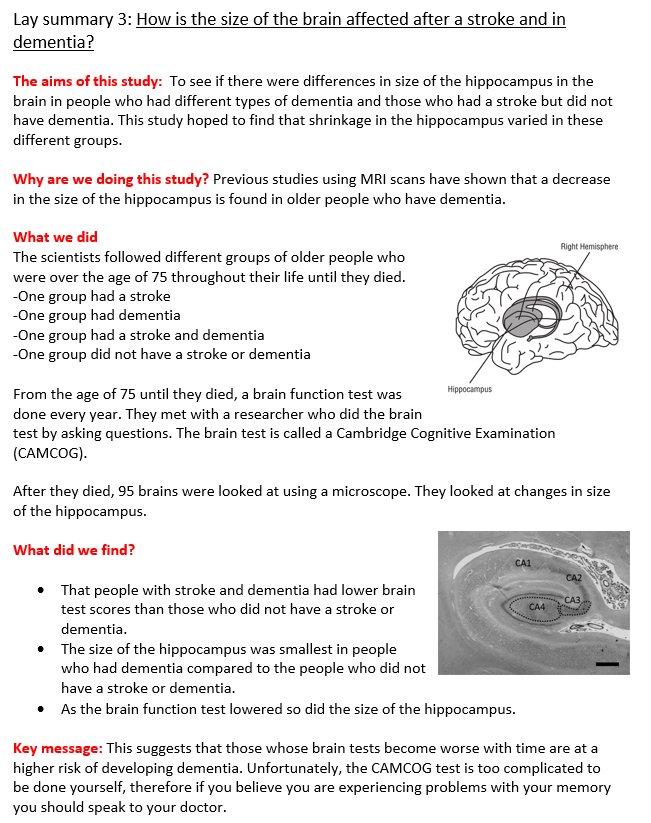

Supplement: Supplementary file 3 — Second draft of lay summary 3 – Completed after final focus group 1- Tuesday 16th February 2016. (ZIP 166 kb) [file 40900_2017_66_MOESM3_ESM.zip › additional file 3/Second draft of lay summary - side AR2.png]

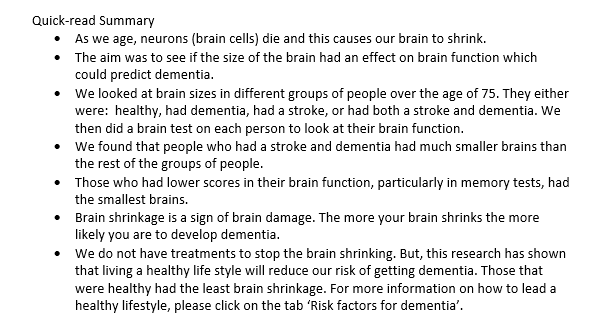

Supplement: Supplementary file 3 — Second draft of lay summary 3 – Completed after final focus group 1- Tuesday 16th February 2016. (ZIP 166 kb) [file 40900_2017_66_MOESM3_ESM.zip › additional file 3/Second draft of lay summary - side BR2.png]
