# Supplementary material for: “Is all the stuff about neurons necessary?” The development of lay summaries to disseminate findings from the Newcastle Cognitive Function after Stroke (COGFAST) study
Source: Res Involv Engagem. 2017 Sep 11;3:18. doi: 10.1186/s40900-017-0066-y (PMC5611656; doi:10.1186/s40900-017-0066-y)
Supplement: Supplementary file 4 — Final version of lay summary 3 – Completed after focus group 2 – 26th February 2016. (PDF 429 kb) [file 40900_2017_66_MOESM4_ESM.pdf]

Newcastle University

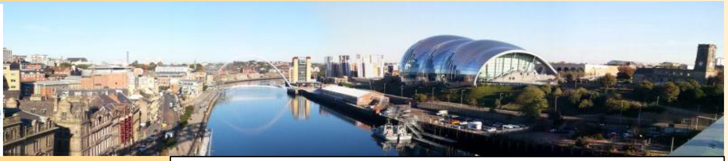

Cognitive Function after Stroke Project

How is the size of the brain affected after a stroke and in dementia?

Quick-read Summary

- As we age, neurons (brain cells) die naturally and this causes our brain to shrink.
- The hippocampus is found in the middle of the brain; its function is for learning and memory.
- The aim was to see if the size of the hippocampus had an effect on brain function which could predict dementia.
- We looked at hippocampus sizes in different groups of people over the age of 75. They either were: healthy (no dementia or stroke), had dementia, had a stroke, or had both a stroke and dementia. We then did a brain test on each person to look at their brain function.
- We found that people who had a stroke and dementia, had a smaller hippocampus than the other groups of people.
- Those who had lower scores in their brain function, particularly in memory tests, had the smallest brains.
- Hippocampus shrinkage is a sign of brain damage. The more your brain shrinks the more likely you are to develop dementia.
- We do not have treatments to stop the brain shrinking. But, this research has shown that living a healthy life style will reduce our risk of developing dementia. Those that were healthy had the least brain shrinkage.

The aims of this study

To see if there were differences in size of the hippocampus in the brain in people who had different types of dementia and those who had a stroke but did not have dementia. This study aimed to find out if shrinkage in the hippocampus varied in these different groups.

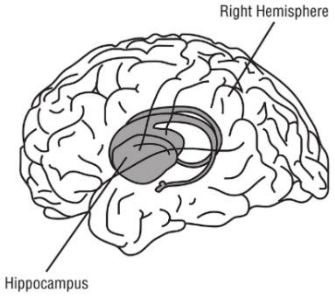

Simplified diagram of the brain

Original paper found here: Gemmell E, Bosomworth H, Allan L, Hall R, Khundakar A, Oakley AE, Deramecourt V, Polvikoski TM, O'Brien JT, Kalaria RN. Hippocampal Neuronal Atrophy and Cognitive Function in Delayed Poststroke and Aging-Related Dementias. *Stroke* 2012, **43**(3), 808-814.

What we did continued…

The scientists followed different groups of older people who were over the age of 75 throughout their life until they died.

-One group had a stroke

-One group had dementia

-One group had a stroke and dementia

-One group did not have a stroke or dementia

From the age of 75 until they died, a brain function test was done every year. They met with a researcher who did the brain test by asking questions. The brain test is called a Cambridge Cognitive Examination (CAMCOG).

After they died, 95 brains were looked at using a microscope. They looked at changes in size of the hippocampus.

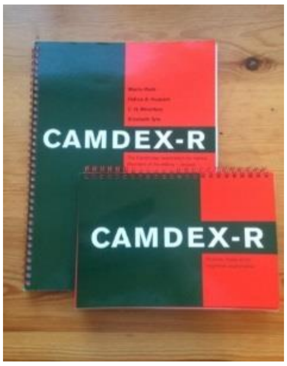

Photo of CAMDEX-R, CAMCOG test found inside

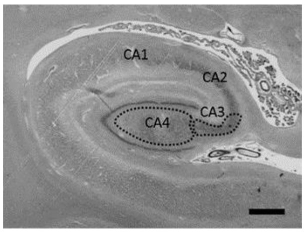

The hippocampus under a microscope

What did we find?

- That people with stroke and dementia had lower brain test scores than those who did not have a stroke or dementia.
- The size of the hippocampus was smallest in people who had dementia compared to the people who did not have a stroke or dementia.
- As the brain function test score lowered so did the size of the hippocampus.

Key message: This suggests that those whose brain tests become worse with time are at a higher risk of developing dementia. Unfortunately, the CAMCOG test is too complicated to be done by yourself. If you believe you have problems with your memory, you should speak to your doctor.

Original paper found here: Gemmell E, Bosomworth H, Allan L, Hall R, Khundakar A, Oakley AE, Deramecourt V, Polvikoski TM, O'Brien JT, Kalaria RN. Hippocampal Neuronal Atrophy and Cognitive Function in Delayed Poststroke and Aging-Related Dementias. *Stroke* 2012, **43**(3), 808-814.
